# Greater residential greenness is associated with reduced epigenetic aging in adults

**DOI:** 10.1038/s41598-024-82747-3

**Published:** 2025-01-28

**Authors:** Andrey I. Egorov, Shannon M. Griffin, Jo Klein, Wei Guo, Jennifer N. Styles, Jason Kobylanski, Mark S. Murphy, Elizabeth Sams, Edward E. Hudgens, Timothy J. Wade

**Affiliations:** 1 Office of Research and Development, United States Environmental Protection Agency, 104 Mason Farm Rd., Chapel Hill, NC 27514 USA; 2 Office of Research and Development, United States Environmental Protection Agency, Cincinnati, OH USA; 3 Zymo Research Corp., Irvine, CA USA; 4 Department of Environmental Sciences and Engineering, Gillings School of Global Public Health, University of North Carolina, Chapel Hill, NC USA; 5 U.S. EPA National Geospatial Support Team (NGST), Research Triangle Park, NC USA

**Keywords:** Epigenetic age, DNA methylation, Residential greenness, Tree cover, Vegetated land cover, Green spaces, DNA, Predictive markers, Environmental impact

## Abstract

Potential pathways linking urban green spaces to improved health include relaxation, stress alleviation, and improved immune system functioning. Epigenetic age acceleration (EAA) is a composite biomarker of biological aging based on DNA methylation measurements; it is predictive of morbidity and mortality. This cross-sectional study of 116 adult residents of a metropolitan area in central North Carolina investigated associations between exposure to residential green spaces and EAA using four previously developed epigenetic age formulas. DNA methylation tests of white blood cells were conducted using Illumina MethylationEPIC v1.0 assays. EAA values were calculated as residuals from the linear regression model of epigenetic age on chronological age. Residential greenness was characterized using tree cover, total vegetated land cover, and normalized difference vegetation index (NDVI) data. An interquartile range (IQR) increase in distance-to-residence weighted average greenness within 500 m of residence was consistently associated with a reduced EAA adjusted for sociodemographic covariates, smoking status, white blood cell fractions, and the two-dimensional spline function of geographic coordinates. The reduction in the EAA estimates for the four EAA measures ranged from − 1.0 to − 1.6 years for tree cover, from − 1.2 to − 1.5 years for vegetated land cover, and from − 0.9 to − 1.3 years for the NDVI; 11 of the 12 associations were statistically significant (*p* < 0.05). This study produced new evidence linking reduced epigenetic aging to greater greenness near residences.

**Supplementary Information:**

The online version contains supplementary material available at 10.1038/s41598-024-82747-3.

## Introduction

Methylation of a cytosine nucleotide followed by guanine, the arrangement known as a CpG site, affects the functions of regulatory elements and modifies gene expression. Biological aging in humans is associated with DNA methylation changes at multiple genomic sites^1^. Various statistical formulas have been developed to calculate epigenetic age (EA) based on DNA methylation data^2–4^. Increased EA relative to chronological age, which is known as epigenetic age acceleration (EAA), has been linked to increased risks of cardiovascular diseases, cancer, diabetes, and mortality^3,5–8^. Factors that have been associated with increased EAA include exposure to social stressors, low socioeconomic status, unhealthy lifestyle, obesity, and exposure to air pollution^3,4,9–12^. Exercise, healthy lifestyle, and other health-promoting factors have been linked to reduced EAA^3,13^.

Contact with green spaces and natural environments has been linked to clinical health benefits, including reduced morbidity and mortality^14–17^. Residential greenness has also been linked to a reduced biomarker-based measure of physiological dysregulation known as allostatic load (AL)^18–20^. AL is a composite measure of subclinical health impacts that is predictive of systemic diseases and mortality^21^.

To our knowledge, only two previous studies have shown associations between residential greenness and EAA measures^22,23^. The results of those studies were rather inconsistent: first, there were different patterns of effect modifications by socioeconomic status (stronger effects in individuals with low SES in one study and in individuals with high SES in another study); second, there were inconsistent spatial patterns with stronger effects associated with small buffer sizes in one study and very large buffer sizes in another study. In addition, beneficial effects were not observed for all EAA measures employed in each study.

The present study aimed to characterize associations between various residential greenness measures and four alternative EAA measures in the same population where beneficial effects of residential greenness on AL were demonstrated previously^20^.

## Methods

### Study design

The present study involved a subset of adult individuals who participated in an observational community study in the Raleigh-Durham-Chapel Hill, North Carolina metropolitan area. The study protocol was approved by the Institutional Review Board at the University of North Carolina in Chapel Hill (IRB # 17-1981). This research was performed in accordance with the Declaration of Helsinki and other relevant guidelines and regulations. Informed consent was obtained from all participants prior to data collection. Participants were recruited through advertisements in local newspapers and websites. The enrollment criteria included age at least 18 years and continuous residence at the same address for at least three years prior to enrollment. Individuals who were unable to communicate in English, move around without assistance, or provide blood samples were excluded. Data collection was conducted at the U.S. Environmental Protection Agency (US EPA) Human Studies Facility in Chapel Hill, NC. A baseline survey from November 2017 through July 2018 involved anthropometrical measurements and a questionnaire with questions on sociodemographic characteristics, smoking habits, chronic diseases, behavioral patterns such as daily time spent outdoors, sleeping habits, and physical activities, as well as a short version (21 questions) of the standard Depression, Anxiety and Stress Survey (DASS)^24^. The baseline serum samples were tested for immunoglobulin G (IgG) responses to three common chronic infections: cytomegalovirus (CMV), *Helicobacter pylori*, and *Toxoplasma gondii* using commercially available enzyme-linked immunosorbent assay (ELISA) kits as described previously^20^. A subset of baseline study participants (*n* = 117) who continued residing at the same address then provided one whole blood sample per person for DNA methylation tests from November 2018 through July 2019, approximately one year after the baseline survey. The samples were stored at − 80 °C until overnight shipment on dry ice to a contractor laboratory at the University of Minnesota Genomics Center (UMGC, Minneapolis, MN).

### Residential greenness data

This analysis used three types of cross-sectional residential greenness data around each residence: tree cover proportion, total vegetated land cover (trees and herbaceous vegetation combined) proportion, and normalized difference vegetation index (NDVI) – remote sensing data reflecting green vegetation density during the peak vegetative season (summer months in North Carolina). NDVI values can vary from − 1 to 1 with negative values close to − 1 corresponding to water bodies, values near 0 corresponding to bare land or rocks, and values close to 1 corresponding to dense green vegetation.

The residential addresses of the study participants were geocoded using ArcGIS desktop software and the ArcGIS World Geocoding Service (Esri Inc., Redlands, CA, USA). Tree cover and vegetated land cover measures were calculated using QGIS Desktop 3.8.1 with GRASS 7.6.1. Land cover data for the Durham-Chapel Hill urban area with a 1-m resolution, including tree cover and total vegetated cover were derived from the US EPA EnviroAtlas database (https://www.epa.gov/enviroatlas)^25^; similar 1-m resolution land cover data for the city of Raleigh and its vicinities were derived from a land cover database provided by Dr. Melissa McHale (currently at the University of British Columbia). The latter dataset has been previously used in environmental studies^26,27^. It was also validated against the EnviroAtlas data in the partial overlap area; correlations between the two sets of tree cover and vegetated land cover measures derived from the two datasets were 0.95 each as described previously^20,28^. NDVI values were calculated in ArcGIS Pro using 10-m resolution Sentinel-2 satellite images for August 2018 extracted from the Copernicus Data Space Ecosystem (https://dataspace.copernicus.eu).

Based on previous assessments of residential greenness exposure, the primary exposure metrics in the present study were distance-to-residence weighted average greenness measures within a 500 m buffer, allowing a decrease in effect with increasing distance from residences^19,20^. These greenness measures were calculated in two steps: (1) average greenness values were calculated for ten concentric 50 m-wide annuli, from 0 to 50 m to 450–500 m; and (2) annulus-specific data were used to calculate a weighted average value using annulus-specific weights declining with increasing distance from the residence. These weights (Supplemental Fig. 1) were derived from the cumulative probability distribution of an exponential decay function with a rate parameter λ = 0.0025 as described previously^19,20^. In addition, a sensitivity analysis of buffer sizes was conducted using average (non-weighted) greenness measures within buffer sizes of 50, 100, 300, 500, and 1000 m as was done previously in the analysis of the effects of residential greens on allostatic load^20^.

### DNA extraction and DNA methylation tests

DNA extraction and DNA methylation tests were conducted in two batches in 2020 and 2022. DNA was extracted using QIAamp QIAcube HT kits (Qiagen, Germantown, Maryland, USA). To assess variability due to the assay, duplicate aliquots of four samples were processed separately starting from the DNA extraction step. Extracted DNA was quantified using Quant-iT™ PicoGreen™ dsDNA quantitation kits (Invitrogen, Waltham, Massachusetts, USA). All the samples produced > 500 ng of DNA, which was sufficient for methylation tests. Following bisulfite conversion of unmethylated cytosine to uracil, DNA methylation tests were conducted using Infinium MethylationEPIC BeadChip v1.0 (Illumina, San Diego, California, USA), which produces multiplexed measurements at more than 865,000 CpG sites^29^. The methylation level of each CpG site was characterized using the probe’s β value, which reflects the relative intensity of the methylated signal, with values ranging from zero (completely nonmethylated) to one (fully methylated).

### DNA methylation data processing

DNA methylation data were processed and cleaned in R using the Bioconductor package ***minfi*** version 1.42.0^30,31^. In the Infinium EPIC v1.0 assay, methylation levels are measured using Type I or Type II probes, which exhibit different behaviors necessitating a data normalization step. The accuracy of probes is also influenced by the presence of single nucleotide polymorphisms (SNPs) and cross-hybridization. In addition, specific samples may fail analysis if a large proportion of probes fail or if there is a mismatch in the reported sex vs. the predicted sex^29,32^.

Data preprocessing was performed using the functional normalization method^33^. Sample quality was assessed using detection *p* values for probes, which were calculated by contrasting the measured signal intensity in samples and background signal intensity in negative controls. Samples were considered invalid if *p* values were higher than 0.05 for at least 10% of all probes (no samples were excluded for this reason). One sample was removed from further analysis because the participant’s sex determined using the ***minfi*** did not match the recorded sex, reducing the number of participants to 116.

Low-quality probes failing a detection *p* value threshold of 0.05 in at least 10% of the samples (1,189 probes), probes on the X and Y chromosomes (19,245 probes), cross-reactive probes (26,353 probes), and probes containing a SNP as determined by ***minfi*** (28,192 probes) were filtered out prior to analysis leaving 790,880 probes or 91.3% of the original population of 865,859 probes. Data processing also involved the ComBat empirical Bayesian batch-correction approach^34^, which was implemented using the R package ***sva***. As many age-associated DNA methylation changes in human immune cells are cell type-specific, EAA measures can also be correlated with cell type counts^35^. In this study, processed DNA methylation data were used to calculate the proportions of six types of white blood cells (WBCs): CD8+ T cells, CD4+ T cells, natural killer cells, B cells, monocytes, and granulocytes. These calculations were conducted in ***minfi*** using Houseman’s method^36^.

### EAA estimation

Further statistical analysis was conducted using SAS version 9.4 (SAS Institute, Cary, NC) software. Four alternative sets of EA values were calculated using the empirical “epigenetic clock” formulas developed by Hannum et al., Horvath, Levine et al., and Li et al.^4,37–40^. Horvath’s EA was developed using a meta-analysis of data for multiple tissues including WBC and utilizing DNA methylation data for approximately 21,000 CpG sites available on both the Infinium Methylation450 and Methylation27 platforms vs. chronological age^38,40^. Hannum’s EA was developed by regressing DNA methylation data from the Methylation450 platform for WBC on chronological age adjusting for covariates, such as sex, body mass index (BMI), diabetes status, and ethnicity^39^. Levine’s EA was also developed using WBC DNA methylation data from the Methylation450 platform through a two-step process involving the development of a phenotypic age index (PhenoAge) using multiple biomarker data, and then regressing DNA methylation data on PhenoAge^37^. Finally, Li’s EA was developed using data from the Methylation450 platform through regressing WBC DNA methylation data on age and adjusting for covariates such as sex, smoking status, alcohol consumption, BMI, and leukocyte type proportions^4^.

Horvath’s, Hannum’s, and Levine’s PhenoAge epigenetic clocks were selected because of their widespread application in research studies^2,3^. Li’s epigenetic clock was included because of its previously demonstrated association with exposure to polycyclic aromatic hydrocarbons (PAHs) in indoor air^4,41^. Formulas for estimating EA values using sets of CpG-specific coefficients were obtained from the corresponding original publications. Some CpG sites that were used in these EA formulas based on previous generation Infinium assays were not included in Infinium EPIC v1.0. In addition, certain CpG sites were removed at the data processing stage as described above. As a result, the total number of CpG sites used in the EA formulas was reduced as follows: Horvath’s EA, from 353 to 334 (94.6%); Hannum’s EA, from 71 to 63 (88.7%); Levine’s PhenoAge EA, from 513 to 511 (99.6%); and Li’s EA, from 239 to 209 (87.4%).

Duplicate samples were used to calculate the mean standard deviations (SD) of the EA values due to the assay. Further statistical analysis utilized mean EA values for duplicate samples.

### Statistical data analysis

All the statistical analyses beyond the DNA methylation data processing stage were conducted using SAS v. 9.4 (SAS Institute, Cary, NC). EAA values were calculated as residuals from linear regression of EA on chronological age. Missing values for race, ethnicity, cytomegalovirus infection status, and household income (Table 1) were imputed using the multiple imputations procedure (SAS procedure ***mi***) based on the discriminant analysis method. Race data were dichotomized as white vs. nonwhite.

**Table 1:** Descriptive statistics of the study population.

Variable	Level	Number	Mean (SD) or percent
Age, years	All	116	57.7 (13.4)
Sex	Male	45	38.8%
	Female	71	61.2%
Race	Black	34	29.3%
	White	74	63.8%
	Asian	0	0.0%
	American Indian	1	0.9%
	Mixed	5	4.3%
	Not reported	2	1.7%
Ethnicity	Hispanic	5	4.3%
	Not Hispanic	111	95.7%
Education	Below high school	0	0.0%
	High school	11	9.5%
	Below 4 years of college	25	21.6%
	Bachelor’s degree	35	30.2%
	Graduate degree	45	38.8%
Annual household income in US dollars	Less than 25,000	16	13.8%
	25,000–49,000	26	22.4%
	50,000–99,000	47	40.5%
	100,000 or more	17	14.7%
	Not reported	10	8.6%
Current smoker	Yes	16	13.8%
	No	100	86.2%
BMI category	Obese	45	38.8%
	Overweight	33	28.4%
	Normal weight	37	31.9%
	Underweight	1	0.9%
Cytomegalovirus	Seropositive	58	50.0%
	Seronegative	57	49.1%
	No data	1	0.9%
*H. pylori*	Seropositive	27	23.3%
	Seronegative	89	76.7%
*T. gondii*	Seropositive	13	11.2%
	Seronegative	103	88.8%
Daily screen time	0–2 h	31	26.7%
	> 2 h	85	73.3%
Difficulties with sleeping	No	61	52.6%
	Yes	55	47.4%
Time outdoors on weekdays	Less than 30 min	17	14.7%
	30 min to 1 h	53	45.7%
	> 1 but less than 3 h	35	30.2%
	3 h or more	11	9.5%

Regression models were developed in two stages. At the first stage, sociodemographic and behavioral covariates for multivariate regression models were selected using stepwise bidirectional elimination by minimizing the Akaike’s information criterion corrected (AICc). The list of covariates considered for the final models is presented in Supplemental Table 1. It included sex, age, race, ethnicity, household income, education, marital status (married vs. not married), height, weight, body mass index (BMI), waist-to-hip ratio (WHR), consumption of prefabricated sugary drinks (“soda”), tobacco smoking, daily leisure screen time, distance from residence to a major road, gardening, exercising in any setting, exercising in outdoor settings, daily time spent outdoors, DASS depression, anxiety, and stress scores, diagnoses of cardiovascular disease, diabetes, and depression, seropositivity to CMV, *H. pylori*, and *T. gondii*, count of chronic infections (number of seropositive results), and proportions of WBC types (B cells, CD4+ T cells, CD8+ T cells, granulocytes, monocytes, and natural killer cells). Seropositivity to chronic infections was considered because of previous reports of detrimental impacts on the EAA of *H. pylori* and CMV infections^42–44^. Smoking status was included in all models by design due to its previously reported associations with EAA^41^. As waist-to-hip ratio (WHR) values are sex-specific, sex had to be included along with the WHR. Model selection was repeated for each combination of greenness exposure measure and EAA index. To facilitate comparisons of effect estimates, sociodemographic and behavioral covariates that were selected for at least one model were included in all regression models.

At the second stage, spatial autocorrelation analysis of residuals from the first stage regression models (not including greenness variables) was conducted using SAS procedure ***variogram***. Autocorrelation was assessed using Moran’s I coefficient and Geary’s C ratio^45,46^. This analysis produced evidence of a spatial pattern in Horvath’s EAA not explained by covariates. Further multivariate regression analysis for all EAA measures was conducted using generalized additive models (GAMs) that included, in addition to covariates selected at the previous stage, a two-dimensional spline (“thin-plate spline”) function of geographic coordinates. The same approach with adjusting for a thin-plate spline of coordinates was used for analysis of associations of greenness with allostatic load described previously^19,20^.

An exploratory analysis of potentially nonlinear associations between residential greenness and EAA measures was conducted using GAM multivariate regression models described above and a spline function of greenness data. When a departure from linearity was not detected, further analysis was conducted using a GAM model with a linear term for greenness exposure. When a statistically significant departure from linearity (a statistically significant detrended spline function) was detected, further analysis was conducted using two approaches: (1) GAM models with a linear term for greenness exposure; and (2) GAM models where greenness exposure was modeled using two linear terms with a common intercept at the median greenness value set as the inflection point - the approach known as piecewise linear regression^47^. The piecewise models produced two different linear slopes for greenness values below and above the inflection point.

GAM models with a linear term for greenness were used in a stratified analysis and analysis of interactions to explore potential effect modifications by dichotomized race and education variables; the latter variable was dichotomized for this analysis as no undergraduate degree vs. bachelor’s or graduate degree. Regression models for interaction effects included a binary variable for race or education and a product of this binary variable and a greenness variable.

## Results

### Descriptive statistics

Valid DNA methylation data were produced for 116 of 117 individuals (99.1%); one person was removed at the data processing stage. The mean age of the remaining participants was 57.7 years, ranging from 24.8 to 85.0 years (Table 1). Young adults were underrepresented, possibly because they were less likely to reside at the same address for three or more years (an enrollment criterion) than older individuals. Females comprised 61.2% of the study sample. White (63.8%) and black individuals (29.3%) formed the two largest racial groups. A total of 70.0% of participants had bachelor’s or graduate level degrees. The prevalence of smoking was 13.8% and the prevalence of obesity (BMI ≥ 30 kg/m^2^) was 38.8%.

The greenness data are summarized in Supplemental Table 2. The mean distance-to-residence weighted tree cover proportion within 500 m of residences was 0.60 (range 0.10–0.89) while the mean distance-weighted total vegetated land cover proportion was 0.74 (range 0.25–0.95). The mean distance-weighted NDVI was 0.53 (range 0.27–0.69). The data on average non-weighted greenness measures for buffers of different sizes showed that variability in greenness declined with an increasing buffer size. The greatest inter-quartile range (IQR) values were observed for the 50 m buffer, and the smallest were observed for the 1000 m buffer.

The mean standard deviations of the EA values due to the assay calculated from the paired sample data were 0.8 years (range 0.4–1.5 years) for Hannum’s EA, 1.6 years (range 1.0–2.1 years) for Horvath’s EA, 1.6 years (0.2–3.3) for Levine’s PhenoAge EA, and 2.1 years (1.4–3.2 years) for Li’s EA. The coefficients of determination (R^2^ values) from the regression of EA on chronological age were, in decreasing order, 0.88 for Li’s EA, 0.85 for Hannum’s EA, and 0.74 for Horvath’s EA and PhenoAge EA each. As expected for residuals from a linear regression on age, all mean EAA values were close to zero. The EAA ranges (standard deviations) were − 11.1 to 12.4 (3.7) years for Hannum’s EAA, − 7.7 to 29.4 (4.3) years for Horvath’s EAA, − 8.5 to 20.3 (4.9) years for Levine’s PhenoAge EAA, and − 9.0 to 12.7 (3.6) years for Li’s EAA.

Descriptive statistical analysis demonstrated that correlations between alternative EAA measures ranged from *r* = 0.42 for Levine’s PhenoAge EAA vs. Li’s EAA to 0.64 for Horvath’s vs. Li’s EAA (Supplemental Table 1). All four EAA measures were negatively correlated with greenness measures; the greatest number of significant negative correlations with various greenness measures was observed for Li’s EAA. The height of the participants was significantly positively correlated with all four EAA measures. Three significant negative correlations were observed between educational attainment (ordinal variable) and EAA measures. Sex-specific waist-to-hip ratio (WHR) values were positively correlated with EAA measures. High soda consumption (three or more glasses per day) was significantly positively correlated with all four EAA measures. Correlations between exercising (in any setting and in an outdoor setting only) and EAA measures were negative but not statistically significant. Gardening was significantly negatively correlated with all four EAA measures. *T. gondii* seropositivity and the number of chronic infections (an ordinal variable with values from 0 to 3) were significantly positively correlated with Li’s and Horvath’s EAA measures.

### Associations between residential greenness and EAA

The final multivariate regression models for all the EAA measures included a greenness variable, dichotomized race (white vs. nonwhite), sex, WHR, height, smoking status, and two-dimensional spline function of geographic coordinates. Variables for WBC types were selected separately for each EAA measure (Table 2). Model building results for WBC variables were generally consistent with the observed correlations between EAA measures and WBC proportions (Supplemental Table 1). Age, ethnicity, exercise, education, income, marital status, gardening, soda consumption, BMI, DASS component scores, distance to roads, chronic diseases, and seropositivity to chronic infections did not satisfy the criteria for inclusion in final multivariate models described in the “Methods” section.

**Table 2:** Multivariate generalized additive regression models for associations between greenness measures (distance-weighted tree cover, vegetated land cover, and NDVI within 500 m of residence) and EAA measures.

Parameter	Hannum’s EAA		Horvath’s EAA		Levine’s PhenoAge EAA		Li’s EAA	
	Effect estimate (95% CL)	*p* value	Effect estimate (95% CL)	*p* value	Effect estimate (95% CL)	*p*-value	Effect estimate (95% CL)	*p* value
A. Tree cover								
Tree cover (per IQR increase)	− 1.4 (− 2.3; − 0.5)	0.003	− 1.0 (− 2.2; 0.1)	0.09	− 1.5 (− 2.8; − 0.2)	0.02	− 1.6 (− 2.6; − 0.6)	0.002
White race	1.0 (− 0.4; 2.3)	0.16	0.4 (− 1.3; 2.1)	0.6	0.0 (− 1.9; 1.9)	1.0	1.3 (− 0.1; 2.7)	0.06
Waist-to-hip ratio	8.6 (2.2; 15.1)	0.01	8.6 (0.0; 17.2)	0.05	8.2 (− 0.9; 17.2)	0.08	8.9 (1.7; 16.1)	0.02
Male sex	− 1.1 (− 2.9; 0.8)	0.25	− 0.5 (− 3.0; 2.0)	0.7	− 0.8 (− 3.5; 1.8)	0.5	− 1.3 (− 3.4; 0.8)	0.2
Height (per 1 cm)	0.1 (0.0; 0.2)	0.01	0.1 (0.0; 0.2)	0.1	0.1 (0.0; 0.2)	0.1	0.1 (0.0; 0.2)	0.08
Smoker	1.2 (− 0.5; 2.9)	0.17	1.8 (− 0.5; 4.1)	0.1	1.2 (− 1.1; 3.6)	0.3	1.3 (− 0.7; 3.2)	0.2
CD4+ T cells*	− 1.2 (− 2.4; 0.0)	0.05	− 0.9 (− 2.3; 0.4)	0.2				
CD8+ T cells*							1.3 (− 0.1; 2.8)	0.08
Granulocytes*	0.9 (0.1; 1.7)	0.04			1.7 (0.7; 2.7)	0.001		
Monocytes*					3.5 (0.7; 6.2)	0.01		
Spline of coordinates	NA	0.0001	NA	0.02	NA	< 0.0001	NA	0.2
B. Vegetated land cover								
Vegetated cover (per IQR increase)	− 1.3 (− 2.2; − 0.4)	0.01	− 1.2 (− 2.4; 0.0)	0.04	− 1.5 (− 2.8; − 0.2)	0.02	− 1.4 (− 2.4; − 0.3)	0.009
White race	0.9 (− 0.4; 2.3)	0.2	0.4 (− 1.3; 2.1)	0.7	− 0.1 (− 1.9; 1.8)	0.9	1.3 (− 0.2; 2.7)	0.08
Waist-to-hip ratio	9.2 (2.8; 15.6)	0.01	9.1 (0.6; 17.6)	0.04	8.8 (− 0.3; 17.8)	0.06	9.6 (2.3; 16.8)	0.01
Male sex	− 1.2 (− 3.1; 0.7)	0.2	− 0.6 (− 3.0; 1.9)	0.7	− 0.9 (− 3.6; 1.8)	0.5	− 1.5 (− 3.6; 0.6)	0.2
Height (per 1 cm)	0.1 (0.0; 0.2)	0.01	0.1 (0.0; 0.2)	0.1	0.1 (0.0; 0.2)	0.1	0.1 (0.0; 0.2)	0.1
Smoker	1.3 (− 0.4; 2.9)	0.1	1.8 (− 0.4; 4.1)	0.1	1.3 (− 1.1; 3.6)	0.3	1.3 (− 0.6; 3.3)	0.2
CD4+ T cells*	− 1.2 (− 2.4; 0.0)	0.06	− 1.0 (− 2.3; 0.4)	0.2				
CD8+ T cells*							1.3 (− 0.1; 2.8)	0.07
Granulocytes*	0.9 (0.1; 1.7)	0.03			1.7 (0.8; 2.7)	0.001		
Monocytes*					3.3 (0.6; 6.0)	0.02		
Spline of coordinates	NA	< 0.0001	NA	0.02	NA	< 0.0001	NA	0.4
C. NDVI								
NDVI (per IQR increase)	− 0.9 (− 1.7; − 0.2)	0.02	− 1.1 (− 2.1; − 0.1)	0.03	− 1.3 (− 2.3; − 0.2)	0.02	− 1.2 (− 2.1; − 0.3)	0.01
White race	0.9 (− 0.5; 2.2)	0.20	0.4 (− 1.2; 2.1)	0.6	0.0 (− 1.9; 1.9)	1.0	1.2 (− 0.2; 2.7)	0.08
Waist-to-hip ratio	8.4 (1.9; 15.0)	0.01	8.3 (− 0.2; 16.8)	0.06	7.8 (− 1.3; 16.9)	0.09	8.8 (1.5; 16.1)	0.02
Male sex	− 1.3 (− 3.2; 0.6)	0.18	− 0.6 (− 3.1; 1.8)	0.6	− 1.0 (− 3.6; 1.7)	0.5	− 1.5 (− 3.6; 0.5)	0.1
Height (per 1 cm)	0.1 (0.0; 0.2)	0.005	0.1 (0.0; 0.2)	0.1	0.1 (0.0; 0.2)	0.1	0.1 (0.0; 0.2)	0.07
Smoker	1.2 (− 0.5; 2.9)	0.16	1.8 (− 0.4; 4.0)	0.1	1.2 (− 1.1; 3.6)	0.3	1.3 (− 0.7; 3.2)	0.2
CD4+ T cells*	− 1.2 (− 2.4; 0.1)	0.06	− 1.0 (− 2.4; 0.3)	0.1				
CD8+ T cells*							1.4 (− 0.1; 2.8)	0.07
Granulocytes*	1.0 (0.1; 1.8)	0.02			1.8 (0.8; 2.7)	0.0004		
Monocytes*					3.4 (0.7; 6.1)	0.01		
Spline of coordinates	NA	0.01	NA	0.001	NA	< 0.0001	NA	0.05

*Effect estimates per 10% increase in WBC type fraction.

All associations between greenness variables and EAA measures were indicative of beneficial effects (reduced EAA associated with increased greenness). Overall, the greatest effect size with the smallest *p* value was observed for the association between distance-weighted tree cover and Li’s EAA: an IQR increase in tree cover was associated with − 1.6 (− 2.6; − 0.6) years change in EAA after adjusting for covariates. The only non-significant effect of − 1.0 (− 2.2; 0.1) years was observed for the association between tree cover and Horvath’s EAA (Table 2) and eleven of the twelve associations (92%) were statistically significant (*p* < 0.05). Among the covariates, greater WHR, greater height, and smoking were associated with increased EAA in all the models. Male sex tended to be associated with a reduced EAA after adjusting for the WHR (paired covariate). White race tended to be associated with increased EAA with the strongest effects on Li’s EAA.

**Fig. 1:**
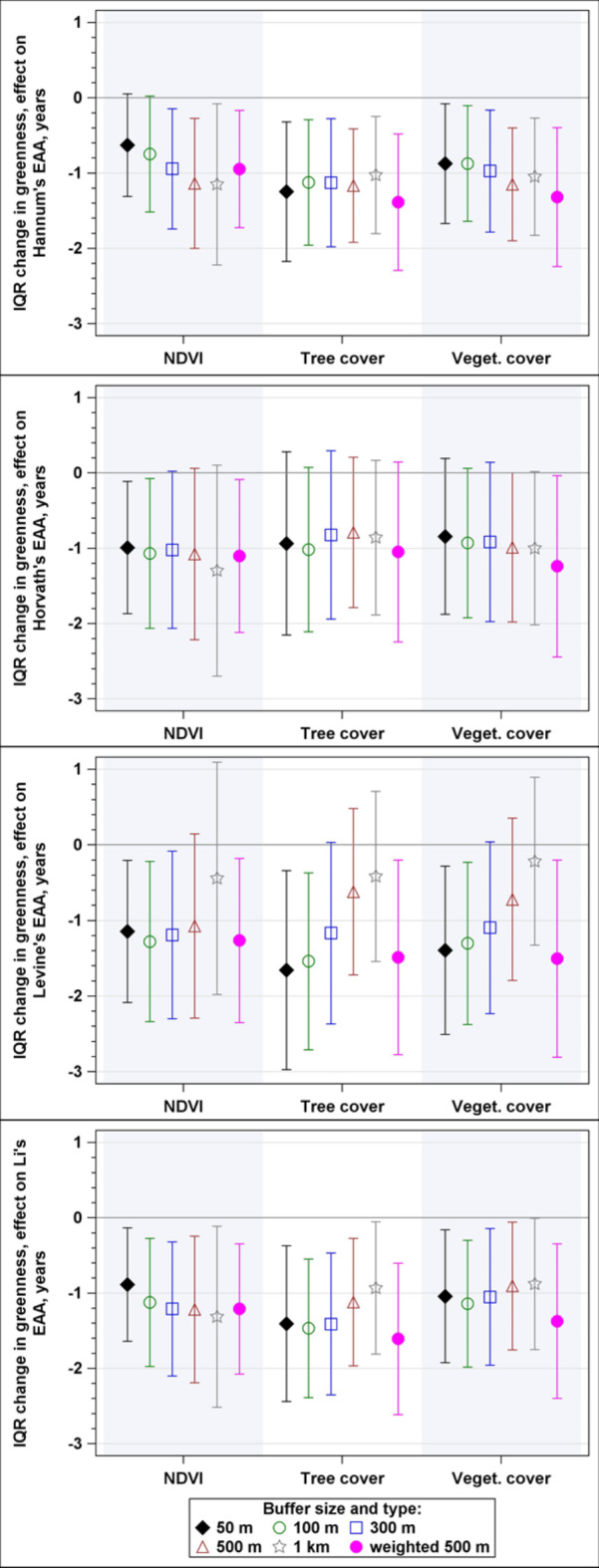
Associaitons between greeness measures and EAA: multiplicative effect estimates with 95% confidence intervals adjusted for race, sex, height, WHR, smoking status, and two-dimensional spline of coordinates.

According to the sensitivity analysis of the buffer size and greenness data aggregation approaches (Fig. 1), 49 of the 72 (68%) associations were statistically significant. Similar proportions of statistically significant associations were observed for the NDVI (17/24, 71%), tree cover (63%) and vegetated land cover (71%). Among the EAA measures, the greatest proportions of significant associations were observed for Li’s EAA (18/18, 100%), followed by Hannum’s EAA (90%), Levine’s PhenoAge EAA (56%), and Horvath’s EAA (28%). Among the non-weighted average greenness measures, the greatest proportions of significant associations were observed for the 50 m and 100 m buffers (9/12, 75.0% each), followed by the 300 m and 500 m buffers (58% each), and 1000 m buffer (50%).

Analysis of departures from linearity of associations between distance-weighted residential greenness measures within a 500 m buffer and EAAs produced evidence of nonlinearity in two models for tree cover and vegetated land cover vs. Hannum’s EAA (Supplemental Table 3). Further piecewise regression analysis of these nonlinear associations demonstrated a consistent pattern with a flat slope at greenness values below the median and a significant inverse association between greenness and EAA for greenness values above the median (Supplemental Table 3).

Results of stratified regression analysis are presented in Supplemental Fig. 2. This analysis did not produce conclusive evidence of differential effects of residential greenness on EAA in white vs. non-white individuals. Although the observed beneficial effects of greenness on Hannum’s and Horvath’s EAA measures were pronounced (statistically significant or almost significant) in white individuals only, the confidence intervals in the two race strata overlapped substantially. Regression analysis stratified by education (individuals without college degree vs. those with undergraduate or graduate degree) also did not reveal any evidence of effect modifications. Further analysis confirmed that all interaction effects of race and greenness, and education and greenness were not statistically significant with *p* values ranging from 0.11 (for the interaction effect of vegetative land cover and race on Horvath’s EAA) to 0.98 (Supplemental Table 4).

## Discussion

This study demonstrated consistent associations between three alternative residential greenness measures (tree cover, vegetated land cover, and NDVI) and reduced epigenetic aging using four previously developed epigenetic clocks^4,37–39^. These findings contribute to the understanding of biological pathways linking residential greenness to improved health, reduced systemic morbidity, and increased life span. These results build upon previously reported associations between residential greenness and reduced allostatic load in the same area^18–20^.

This study used the same distance-to-residence weighted greenness measures within a 500 m buffer which were previously shown to produce the strongest associations with the allostatic load^19,20^. Here, this approach, allowing an exponential decay of effects with increasing distance, once again produced stronger associations with the health outcome variables than non-weighted average greenness measures.

According to the sensitivity analysis of buffer sizes using non-weighted average greenness measures within the 50 m to 1000 m buffers, the strongest effects were found for the 100 m and 50 m buffers. These findings are consistent with our assumption that the main pathways linking residential greenness to improved health are stress alleviation and relaxation leading to improved functioning of neuroendocrine and immune functions, reduced physiological dysregulation, and reduced epigenetic aging. Previous research comparing various buffer sizes demonstrated the strongest associations between residential NDVI values and improved mental health for 100 m buffer, improved perceived health for 250 to 500 m buffers, and improved physical activity for 500 m buffer^48^.

To our knowledge, only two previous studies, one in Australia and another in the United States, have explored associations between residential greenness and EAA^22,23^. The average NDVI values in our study (e.g., 0.54 within a 1 km buffer) were similar to the NDVI values in the Australian study (0.56 within a 1 km buffer), but higher than those reported in the US study (0.38 within a 5 km buffer). The Australian study demonstrated that greater NDVI and greater enhanced vegetation index (EVI) values in the vicinity of residences were significantly inversely associated with one of the four EAA measures used only^22^. According to their stratified analysis, the associations were stronger in the low socioeconomic status stratum. Among the four buffer sizes tested (range from 300 m to 2 km), the strongest effects were observed for the 300 m buffer and the weakest effects were observed for the 2 km buffer. In contrast with the findings presented in this manuscript, there were practically no effects of greenness on Levine’s PhenoAge EAA in the Australian study.

A US study detected significant associations between NDVI and two EAA measures out of three alternative EAA measures tested including a significant association with Levine’s PhenoAge EAA^23^. According to their stratified analysis, the associations were stronger in the higher socioeconomic status group – in contrast to the Australian study findings. Among the buffer sizes tested from 250 m to 5 km, significant associations were observed only for a 5 km buffer, which conflicts with the results of the Australian study and the findings presented here.

These discrepancies in findings might be due to differences in study methodologies and populations. The types of housing, residential greenery, climate, and human behavior might differ substantially in various geographic regions affecting associations between residential greenness and epigenetic aging. More research in different populations is needed to elucidate the spatial patterns of associations between urban green spaces and biological aging as well as underlying mechanisms and pathways with potential differential effects in various socioeconomic and demographic strata.

While the two previous studies produced discrepant findings on the effect modifications by socioeconomic status, the present study found no evidence of effect modifications by race or education. However, the relatively small sample size of the present study resulted in the low statistical power of effect modification tests. Therefore, these findings need to be replicated in further research.

Adjusting for WBC type fractions had very little impact on the associations between greenness and EAA measures in the present study. For example, without adjusting for CD8+ T-cells, the effect estimate for Li’s EAA was − 1.7 (− 2.7; − 0.7) years per IQR increase in tree cover (not shown in the “Results” section) compared to the CD8+ adjusted effect estimate of − 1.6 (− 2.6; − 0.6) years, suggesting that confounding by WBC composition was rather weak. This result is consistent with previous findings that also demonstrated a very limited impact of WBC adjustments on the observed associations between exposure to PAHs and EAA^4^.

White race was included in the final regression models because of its association with increased Li’s EAA. This finding appears to be counterintuitive as the majority of nonwhite individuals in this study were black individuals who have a lower average life expectancy than white individuals in the United States^49^. Li’s EAA has been validated only in Caucasian and Asian populations and the authors reported race-specific intercepts for their EA formula^4^. Further validation of this epigenetic clock in populations of African descent is warranted.

Consistent positive associations between adult height and EAA measures presented another unexpected finding. Previous studies have shown that taller adolescents have greater EAA and that greater height-for-age measures in children are associated with increased Levine’s PhenoAge EAA in mid-life^50,51^. Potential associations between growth rates in children and their final height in adulthood as well as EAA in mid-life warrant further investigation.

The positive associations of the WHR with EAA and male sex with EAA are consistent with previous research demonstrating similar effects of obesity and male sex^3,41^. However, the beneficial effects of physical activity on EAA found in some previous studies^3,41^ were not observed in the present study.

Analysis of duplicate samples demonstrated the level of variability in EA values due to the assay (mean standard deviation values from 0.8 to 2.1 years depending on the epigenetic clock as shown in the Results) consistent with previously published findings^52^. These analytical errors in DNA methylation data are likely to be nondifferential with respect to greenness exposure; therefore, they likely biased the observed effects of greenness on EAA towards the null.

Exposure to green spaces has been linked to differential DNA methylation at CpG sites affecting AL pathways and changes in EAA have been reported to precede changes in AL in prospective study settings^53,54^. The present study could not explore the potential association between EAA as a predictor and AL as an outcome using biomarker data from the previous publication^20^ because the blood samples for DNA methylation tests were collected about one year after AL biomarker data. Further research is warranted to elucidate the relationships between EAA and AL measures.

Accelerated epigenetic aging is at least partially reversible, and switching to a healthier lifestyle has been shown to produce beneficial effects on EAA^55^. Further research in prospective settings is warranted to assess the potential effects of lifestyle changes as well as changes in greenness exposure due to moving to a new residence.

## Conclusions

This study produced evidence that exposure to residential greenness is associated with reduced epigenetic aging. This finding builds upon the previously demonstrated inverse association between residential greenness and AL in the same population and further contributes to our understanding of pathways linking urban greenness to improved clinical outcomes.

## Electronic supplementary material

Below is the link to the electronic supplementary material.

Supplementary Material 1

## Data Availability

The datasets generated and analyzed during the current study are not publicly available due to Personally Identifiable Information (PII) on study participants, but are available from the corresponding author on reasonable request. EPA cannot publicly release PII regarding living individuals according to the Privacy Act and the Freedom of Information Act (FOIA). Further information about requesting access to these data for researchers who meet the criteria for access to confidential data is available at https://catalog.data.gov/organization/epa-gov.
