# Supplementary material for: Greater residential greenness is associated with reduced epigenetic aging in adults
Source: Sci Rep. 2025 Jan 28;15:3558. doi: 10.1038/s41598-024-82747-3 (PMC11775256; doi:10.1038/s41598-024-82747-3)
Supplement: Supplementary file 1 — Supplementary Material 1 [file 41598_2024_82747_MOESM1_ESM.pdf]

Greater residential greenness is associated with reduced epigenetic aging in adults

Andrey I. Egorov [a], Shannon M. Griffin [b], Jo Klein [a], Wei Guo [c], Jennifer N. Styles [a, d], Jason Kobylanski [a], Mark S. Murphy [e], Elizabeth Sams [a], Edward E. Hudgens [a], Timothy J. Wade [a]

[a] Office of Research and Development, United States Environmental Protection Agency, Chapel Hill, NC, USA.

[b] Office of Research and Development, United States Environmental Protection Agency, Cincinnati, OH, USA.

[c] Zymo Research Corp., Irvine, CA, USA.

[d] Department of Environmental Sciences and Engineering, Gillings School of Global Public Health, University of North Carolina, Chapel Hill, NC, USA.

[e] U.S. EPA National Geospatial Support Team (NGST), Research Triangle Park, NC, USA.

SUPPLEMENTAL MATERIALS

SUPPLEMENTAL TABLES

Supplemental Table 1. Correlations among EAA measures and predictor variables

Variable	Correlation type	Hannum's EAA	Horvath's EAA	Levine's EAA	Li's EAA
**Epigenetic age acceleration measures**					
Hannum's EAA	Pearson				
Horvath's EAA	Pearson	0.62**			
Levine's EAA	Pearson	0.57**	0.54**		
Li's EAA	Pearson	0.60**	0.64**	0.42**	
**Greenness measures**					
Tree cover (average within 50 m)	Pearson	-0.13	-0.12	-0.15	-0.21*
Tree cover (average within 100 m)	Pearson	-0.13	-0.16	-0.17	-0.24*
Tree cover (average within 300 m)	Pearson	-0.16	-0.15	-0.16	-0.23*
Tree cover (average within 500 m)	Pearson	-0.21*	-0.16	-0.13	-0.21*
Tree cover (average within 1000 m)	Pearson	-0.17	-0.11	-0.06	-0.14
Tree cover (distance-weighted within 500 m)	Pearson	-0.17	-0.16	-0.17	-0.24**
Vegetated land cover (average within 50 m)	Pearson	-0.09	-0.12	-0.15	-0.17
Vegetated land cover (average within 100 m)	Pearson	-0.09	-0.15	-0.16	-0.19*
Vegetated land cover (average within 300 m)	Pearson	-0.14	-0.17	-0.15	-0.18
Vegetated land cover (average within 500 m)	Pearson	-0.19*	-0.19*	-0.11	-0.18
Vegetated land cover (average within 1000 m)	Pearson	-0.16	-0.14	-0.04	-0.13
Vegetated land cover (distance-weighted within 500 m)	Pearson	-0.15	-0.18	-0.16	-0.20*
NDVI (average within 50 m)	Pearson	-0.05	-0.11	-0.09	-0.14
NDVI (average within 100 m)	Pearson	-0.06	-0.11	-0.10	-0.15
NDVI (average within 300 m)	Pearson	-0.13	-0.11	-0.11	-0.15
NDVI (average within 500 m)	Pearson	-0.16	-0.10	-0.07	-0.14
NDVI (average within 1000 m)	Pearson	-0.11	-0.05	0.00	-0.08
NDVI (distance-weighted within 500 m)	Pearson	-0.12	-0.12	-0.10	-0.16
**Covariates considered for multivariable regression analysis**					
Sex (male)	Point biserial	0.30**	0.24**	0.22*	0.11
Education	Kendall	-0.17*	-0.15*	-0.12	-0.17*
Income	Kendall	-0.03	-0.14*	-0.03	-0.20**
White race	Point biserial	0.06	-0.07	-0.07	0.03
Hispanic ethnicity	Point biserial	-0.06	-0.05	-0.06	-0.06
Marital status (married)	Point biserial	-0.10	-0.13	-0.10	-0.12
Height	Pearson	0.38**	0.29**	0.24**	0.18*
Weight	Pearson	0.24**	0.24**	0.26**	0.14
Body Mass Index	Pearson	0.09	0.14	0.19*	0.07
Obesity	Point biserial	0.08	0.08	0.15	0.11
Waist-to-hip ratio in males	Pearson	0.31*	0.21	0.31*	0.10
Waist-to-hip ratio in females	Pearson	0.18	0.21	0.13	0.34**
High soda consumption	Point biserial	0.20*	0.26**	0.20*	0.18*
Current smoker	Point biserial	0.11	0.17	0.11	0.15
Problems with sleeping	Point biserial	-0.02	-0.11	-0.02	-0.16
Daily screen time > 2 hours	Point biserial	-0.02	0.00	0.04	0.12
-Log10(Distance to road)	Pearson	0.04	0.10	-0.03	0.11
Exercising (anywhere)	Point biserial	-0.06	-0.08	-0.05	0.02
Exercising outdoors	Point biserial	-0.04	-0.03	-0.06	-0.01

Variable	Correlation type	Hannum's EAA	Horvath's EAA	Levine's EAA	Li's EAA
Gardening	Point biserial	-0.20*	-0.21*	-0.19*	-0.24*
Time outdoors	Kendall	0.00	0.00	0.03	-0.02
DASS stress score	Pearson	0.09	0.11	0.17	0.15
DASS anxiety score	Pearson	-0.02	0.15	0.15	0.07
DASS depression score	Pearson	0.11	0.01	0.17	0.09
Cardiovascular disease	Point biserial	0.05	-0.01	0.06	0.12
Diabetes	Point biserial	0.05	0.18	0.03	0.09
Depression ever diagnosed	Point biserial	-0.05	0.11	0.05	0.01
Cytomegalovirus seropositive	Point biserial	0.05	0.11	0.02	0.06
Toxoplasma gondii seropositive	Point biserial	0.12	0.24**	0.10	0.27**
Helicobacter pylori seropositive	Point biserial	0.05	0.03	0.12	0.03
Number of chronic infections	Kendall	0.08	0.18*	0.08	0.14*
B cells proportion	Pearson	-0.09	0.04	-0.04	0.09
CD4+ T cells proportion	Pearson	-0.36**	-0.20*	-0.35**	-0.14
CD8+ T cells proportion	Pearson	-0.16	0.11	-0.04	0.17
Granulocytes proportion	Pearson	0.36**	0.05	0.33**	-0.03
Monocytes proportion	Pearson	0.10	0.10	0.20*	0.00
Natural Killer cell proportion	Pearson	0.08	0.04	-0.12	0.08

* $0.01 \leq p < 0.05$;   ** $p < 0.01$

Supplemental Table 2. Summary of greenness measures within 500 m of residence

Greenness measure	Buffer size, m	Data aggregation method	Min	25th pctl	Median	75th pctl	Max	IQR	Mean
Tree cover	50	Mean	0.00	0.28	0.49	0.77	0.99	0.49	0.50
Tree cover	100	Mean	0.00	0.36	0.57	0.73	0.96	0.37	0.53
Tree cover	300	Mean	0.10	0.44	0.57	0.71	0.94	0.28	0.57
Tree cover	500	Mean	0.17	0.49	0.59	0.70	0.90	0.20	0.60
Tree cover	1000	Mean	0.33	0.52	0.61	0.68	0.89	0.16	0.60
Tree cover	500	Distance-weighted	0.10	0.42	0.59	0.71	0.89	0.29	0.57
Vegetated land cover	50	Mean	0.00	0.50	0.69	0.85	1.00	0.36	0.65
Vegetated land cover	100	Mean	0.06	0.55	0.74	0.84	0.97	0.29	0.68
Vegetated land cover	300	Mean	0.29	0.63	0.73	0.84	0.96	0.20	0.72
Vegetated land cover	500	Mean	0.38	0.67	0.74	0.83	0.96	0.16	0.74
Vegetated land cover	1000	Mean	0.52	0.69	0.75	0.81	0.95	0.12	0.75
Vegetated land cover	500	Distance-weighted	0.25	0.59	0.73	0.83	0.95	0.24	0.71
NDVI	50	Mean	0.11	0.45	0.52	0.59	0.70	0.13	0.51
NDVI	100	Mean	0.15	0.46	0.54	0.60	0.70	0.14	0.52
NDVI	300	Mean	0.30	0.48	0.53	0.59	0.70	0.11	0.53
NDVI	500	Mean	0.34	0.48	0.54	0.59	0.69	0.10	0.54
NDVI	1000	Mean	0.37	0.48	0.55	0.59	0.70	0.11	0.54
NDVI	500	Distance-weighted	0.27	0.48	0.54	0.58	0.69	0.11	0.53

Supplemental Table 3. Analysis of non-linearity of associations between grenness (distance-weighted measures within 500 m of residence) and EAA and piecewise linear regression analysis; models adjusted for two-dimensional spline of coordinates, race, sex, height, smoking status, WHR, and WBC type fractions; effect estimates are for an IQR increase in greenness.

EAA	Greenness measure	p value for non-linearity	Greenness below median		Greenness above median	
			Effect estimate (95% CL)	p value	Effect estimate (95% CL)	p value
Hannum's	Tree cover (weighted)	0.03	0.0 (-1.5; 1.5)	1.0	-3.6 (-5.6; -1.5)	0.0006
	Vegetated cover (weighted)	0.004	0.8 (-0.7; 2.3)	0.3	-5.3 (-7.6; -3.1)	<0.0001
	NDVI (weighted)	0.3				
Horvath's	Tree cover (weighted)	0.3				
	Vegetated cover (weighted)	0.2				
	NDVI (weighted)	0.1				
Levine's PhenoAge	Tree cover (weighted)	0.3				
	Vegetated cover (weighted)	0.5				
	NDVI (weighted)	0.7				
Li's	Tree cover (weighted)	0.3				
	Vegetated cover (weighted)	0.5				
	NDVI (weighted)	0.7				

Supplemental Table 4.

EAA	Stratification variable	Reference category	Exposure variable	Interaction Effect (95% CL)	P-value
Hannum's	Race	Non-white	Tree cover	-0.72 (-2.64; 1.19)	0.46
Hannum's	Race	Non-white	Vegetat. cover	-1.02 (-2.89; 0.86)	0.29
Hannum's	Race	Non-white	NDVI	-1.14 (-2.72; 0.43)	0.16
		Non-white			
Horvath's	Race	Non-white	Tree cover	-1.66 (-4.19; 0.88)	0.20
Horvath's	Race	Non-white	Vegetat. cover	-1.97 (-4.39; 0.45)	0.11
Horvath's	Race	Non-white	NDVI	-0.62 (-2.69; 1.45)	0.56
		Non-white			
Levine's PhenoAge	Race	Non-white	Tree cover	1.04 (-1.68; 3.75)	0.45
Levine's PhenoAge	Race	Non-white	Vegetat. cover	0.93 (-1.76; 3.63)	0.50
Levine's PhenoAge	Race	Non-white	NDVI	0.68 (-1.54; 2.91)	0.55
		Non-white			
Li's	Race	Non-white	Tree cover	0.03 (-2.11; 2.17)	0.98
Li's	Race	Non-white	Vegetat. cover	-0.43 (-2.51; 1.66)	0.69
Li's	Race	Non-white	NDVI	-0.45 (-2.23; 1.33)	0.62
Hannum's	Education	No college	Tree cover	0.32 (-1.75; 2.40)	0.76
Hannum's	Education	No college	Vegetat. cover	0.22 (-1.80; 2.24)	0.83
Hannum's	Education	No college	NDVI	-0.31 (-1.82; 1.20)	0.69
Horvath's	Education	No college	Tree cover	-0.19 (-2.96; 2.58)	0.89
Horvath's	Education	No college	Vegetat. cover	0.07 (-2.60; 2.74)	0.96
Horvath's	Education	No college	NDVI	-0.03 (-2.01; 1.94)	0.97
Levine's PhenoAge	Education	No college	Tree cover	1.18 (-1.72; 4.07)	0.43
Levine's PhenoAge	Education	No college	Vegetat. cover	0.80 (-2.04; 3.64)	0.58
Levine's PhenoAge	Education	No college	NDVI	0.36 (-1.75; 2.47)	0.74
Li's	Education	No college	Tree cover	0.15 (-2.15; 2.45)	0.90
Li's	Education	No college	Vegetat. cover	0.16 (-2.10; 2.42)	0.89
Li's	Education	No college	NDVI	-0.69 (-2.37; 0.99)	0.42

SUPPLEMENTAL FIGURES

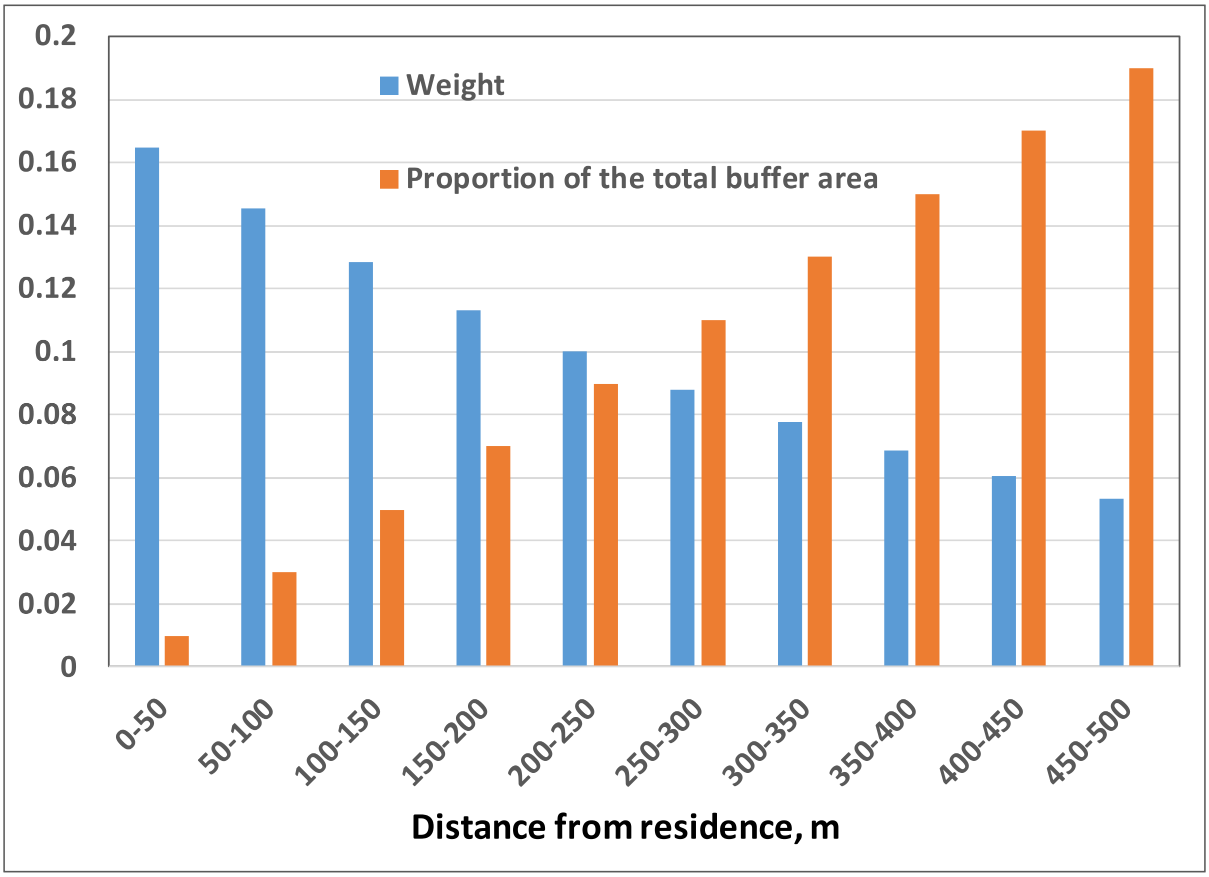

Supplemental Fig. 1. Annulus-specific weights for calculating distance-to-residence-weighted average greenness measures within 500 m buffer using ten 50-meter-wide annuli vs. proportions of annulus areas in the total buffer area. Weights were derived from an exponential decay function and rescaled so that they add up to 1. To calculate non-weighted average greenness, annulus-specific mean greenness values are multiplied by the corresponding area proportions (orange bars); for example, the 0–50 m annulus comprises 1% of the 500 m buffer (7,854 m^2 of 785,398 m^2) while the 450–500 m annulus comprises 19% (149,226 m^2 of 785,398 m^2).

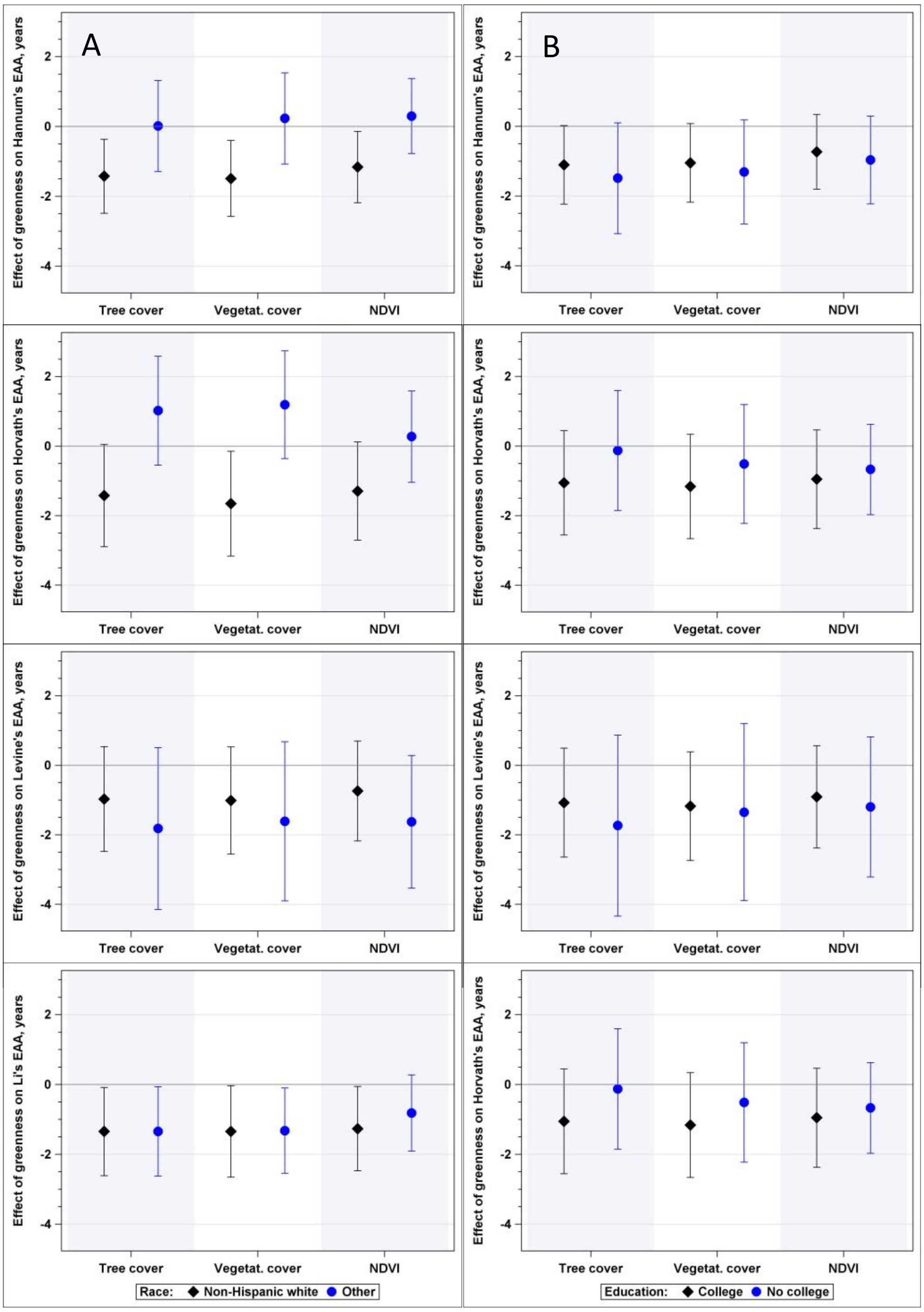

Supplemental Fig. 2. Associations between residential greenness and EAA: A) stratified by race; and B) stratified by education. Effect estimates per an IQR increase in average distance-to-residence weighted greenness measures within 500 m buffer.
